# The gut microbiome in colorectal anastomotic leakage: from mechanisms to precision

**DOI:** 10.3389/fmed.2026.1781458

**Published:** 2026-03-09

**Authors:** Songlin Sun, Feng Long, Bowen Su, Jiahan Chen, Yihuan Luo, Yang Zhong, Guangjun Zhang

**Affiliations:** 1 Second Department of Gastrointestinal Surgery, The Affiliated Hospital of North Sichuan Medical College, National Clinical Key Specialty (General Surgery), Sub-center of National Clinical Research Center for Digestive Diseases, Sichuan Clinical Research Center for Digestive Diseases, Hepatobiliary, Pancreatic and Intestinal Diseases Research Institute, Nanchong, Sichuan, China; 2 Department of Neurology, The Affiliated Hospital of North Sichuan Medical College, Nanchong, Sichuan, China

**Keywords:** anastomotic leakage, biomarkers, collagenolysis, colorectal cancer, dysbiosis, gut microbiome, precision medicine

## Abstract

Anastomotic leakage after curative colorectal cancer resection remains a frequent and severe complication that increases short-term mortality, worsens long-term oncologic outcomes, and places substantial burdens on individuals and health systems despite advances in surgical technique and perioperative care. Emerging evidence redefines anastomotic failure as not only a technical event but also a biologically driven process in which the gut microbiome regulates inflammation, epithelial repair, and barrier integrity at the healing interface. This review summarizes current data on the dual role of the intestinal microbiome in promoting physiological anastomotic healing and driving pathological leakage when perioperative stressors cause dysbiosis. Mechanistic sections describe how a diverse, metabolically active community supports collagen stability through short-chain fatty acid production, immune regulation, and maintenance of mucus and tight junction architecture. In contrast, the enrichment of microbial groups such as *Enterococcus faecalis*, *Fusobacterium nucleatum*, and *Alistipes onderdonkii* together with fungal and viral shifts, has been associated with extracellular matrix degradation and excessive inflammation. Furthermore, the review examines microbiome-related biomarkers for risk assessment, including DNA-based microbial signatures, metabolite profiles, and host immune markers. It also discusses how integrated multi-omics models combined with machine learning may outperform traditional clinical scores for preoperative and early postoperative prediction. Finally, the article critically evaluates perioperative microbiome-directed strategies ranging from dietary prehabilitation and microbial supplementation to selective decontamination and fecal microbiota transplantation, highlighting promising signals, variability of effect, safety considerations, and key methodological limitations that currently prevent routine implementation. In summary, this review addresses three interconnected domains—mechanisms of microbiome-driven anastomotic failure, microbiome-derived biomarkers for risk stratification, and perioperative intervention strategies—underscoring that AL is best understood as a host–microbiome interaction rather than a purely technical failure. This framing offers surgeons and perioperative teams a biologically rational basis for prevention, yet clinical translation will require causal validation, standardized intervention algorithms, and interpretable computational tools embedded into real-world perioperative practice.

## 1 Introduction

Colorectal cancer is one of the most prevalent malignancies worldwide, and surgical resection with anastomotic reconstruction remains the cornerstone of curative treatment. Despite significant advances in operative technique, anesthesia, and perioperative care, postoperative complications continue to affect a substantial proportion of patients and represent a major source of morbidity, mortality, and healthcare expenditure. Among these, anastomotic leakage stands out as the most feared and clinically consequential complication, warranting dedicated investigation into its biological determinants and prevention.

### 1.1 The clinical challenge of anastomotic leakage after colorectal cancer surgery

Colorectal cancer accounts for approximately 1.9 million new diagnoses and nearly 900,000 deaths annually worldwide, representing the third most common cancer and the second leading cause of cancer-related mortality globally (1, 2). Curative-intent colorectal resection depends on a critical biological premise: that the anastomosis will heal faster than contamination, ischemia, and inflammation can damage it. When that premise fails, anastomotic leakage (AL) converts an otherwise controlled oncologic operation into a time-sensitive septic complication, with downstream consequences that extend well beyond the initial admission. Across colorectal procedures, AL typically occurs in roughly 3–15% of cases and may approach 20% after ultra-low anterior resection, making it a persistent, significant threat even in experienced hands (3–5). Its clinical signature—peritoneal infection, sepsis, and occasional multi-organ failure—drives excess short-term mortality (1, 6). Furthermore, the oncologic penalty (higher local recurrence and reduced long-term survival) raises the possibility that a technical complication can alter cancer trajectories (7–9). Re-intervention, prolonged hospitalization, and the prospect of a permanent stoma increase patient-facing morbidity and place a substantial economic burden on health systems (10, 11).

Notably, AL rates have remained persistently unresponsive to “best-practice” refinements, including modern stapling technology, standardized technique, and Enhanced Recovery After Surgery (ERAS)-based perioperative care (12, 13). This stagnation suggests that mechanical considerations—perfusion, tension, and meticulous construction—although necessary, do not fully explain the biological determinants of anastomotic failure (14). Therefore, a clinically meaningful reduction in AL likely requires identifying biological factors that predispose an anastomosis to fail despite technically adequate surgery (15).

### 1.2 The gut microbiome: a key regulator of surgical wound healing

The gut microbiome is increasingly recognized as an active host partner that can regulate immunity, metabolism, and barrier biology—processes that directly govern wound repair (16, 17). In the setting of colorectal surgery, the perioperative period presents a concentrated series of disruptive stressors—bowel preparation, antibiotics, operative stress, ischemia–reperfusion injury, and postoperative fasting—that can trigger abrupt dysbiosis (18, 19). Under these conditions, a community that ordinarily supports mucosal resilience may instead promote inflammation, impair barrier restoration, or enrich organisms that display tissue-disruptive capabilities in experimental settings at the precise moment the anastomosis is most vulnerable (20, 21). Collectively, these perioperative perturbations position the microbiome as both a mechanistic contributor to anastomotic failure and a tractable target for prevention.

### 1.3 Scope and rationale of this review: a tripartite framework of mechanisms, biomarkers, and interventions

Despite rapid growth in microbiome–AL research, the evidence base remains disjointed, with mechanistic insights, candidate biomarkers, and proposed interventions often advancing in parallel rather than coming together into a coherent clinical strategy. Consequently, this review adopts a three-part framework designed to organize the field around the sequence required for translation: mechanistic causality, then risk assessment, then targeted prevention. Specifically, this review investigates microbial and host mechanisms that impair anastomotic healing during dysbiosis, integrates emerging multi-omics predictors for risk assessment, and assesses the clinical viability, safety, and implementation challenges of perioperative microbiome modulation. To support this framework, we conducted a comprehensive literature search in the PubMed database up to 2025. The search strategy included keywords such as “colorectal cancer,” “anastomotic leakage,” “gut microbiome,” “dysbiosis,” “neoadjuvant therapy,” and “perioperative biomarkers.” We prioritized the inclusion of systematic reviews, randomized controlled trials, and pivotal mechanistic studies published within the last decade to ensure the robustness and contemporary relevance of the synthesized evidence. This search ultimately identified more than 200 relevant publications, from which approximately 106 references were selected for inclusion based on their direct relevance to the review’s three-part framework, methodological quality, and recency.

## 2 Role of the microbiome in physiological anastomotic healing

Anastomotic healing occurs through a tightly coordinated cascade—early inflammation that clears debris and primes repair, followed by epithelial proliferation and, ultimately, matrix remodeling that restores tensile strength (16, 22). Within this sequence, a balanced gut microbiome functions less as background flora and more as a biologically active partner that can likely guide repair processes through multiple host–microbe signaling axes (23). Accumulating evidence suggests that commensal communities and their metabolites can regulate postoperative inflammation, sustain the energetic demands of epithelial restoration, reinforce barrier repair, and facilitate angiogenic support at the healing interface, collectively shifting the anastomosis toward durable integrity (2, 24–26).

### 2.1 Precise regulation of the postoperative inflammatory microenvironment

Colorectal anastomosis naturally causes an inflammatory surge that initiates debridement and antimicrobial defense, yet the same response can trigger tissue injury when excessive or prolonged (9, 17). A eubiotic microbiome can likely regulate this postoperative set-point by signaling through pattern recognition receptors (PRRs)—including Toll-like receptors (TLRs)—thereby shaping neutrophil and macrophage recruitment and activation (21, 27). The net effect is a locally controlled, pro-repair inflammatory milieu that supports healing while limiting collateral damage from uncontrolled mediator release (28, 29).

### 2.2 Powering epithelial regeneration and angiogenesis

Rapid epithelial proliferation and migration are metabolically demanding. Microbial fermentation of otherwise indigestible fiber generates short-chain fatty acids (SCFAs), with butyrate serving as a preferred fuel for colonocytes to sustain mucosal restoration (2, 25, 30). Selected commensals (e.g., *Akkermansia muciniphila*) have also been reported to enhance epithelial repair programs, reinforcing the concept that microbial composition and function jointly condition the regenerative response (23). In parallel, the microbiome may regulate angiogenesis—potentially via effects on Paneth cell biology or vascular endothelial growth factor (VEGF)-associated signaling—thereby supporting oxygen and nutrient delivery to the healing interface (26, 31).

### 2.3 Strengthening gut barrier integrity

Because anastomotic healing is, in essence, barrier reconstruction, microbiome-dependent reinforcement of mucus and epithelial junctional architecture may significantly influence early anastomotic resilience (24, 32). By stimulating goblet-cell mucus production, commensal communities can spatially separate luminal bacteria from fragile repairing tissue, reducing opportunities for contact-dependent invasion (23, 33). Moreover, microbial metabolites—particularly butyrate—have been shown to increase tight-junction components (e.g., claudins and zonula occludens-1), tightening the paracellular seal and thereby limiting bacterial translocation and downstream systemic inflammatory sequelae (25, 27, 34).

### 2.4 Key symbiotic bacteria and metabolites in promoting healing

#### 2.4.1 The multidimensional contributions of short-chain fatty acids

Among microbial metabolites, SCFAs—predominantly butyrate, propionate, and acetate—appear to support anastomotic repair through coordinated metabolic and immunologic effects (2, 30). Butyrate supplies colonocytes with a readily oxidizable substrate, thereby supporting the energy demands of epithelial restoration (25, 34). Beyond fueling repair, SCFAs can regulate inflammatory signaling via G-protein–coupled receptors (e.g., GPR43 and GPR109A) and, in the case of butyrate, through histone deacetylase inhibition that likely shifts transcriptional programs toward anti-inflammatory states (35, 36). This immunoregulatory effect is further reinforced by SCFA-associated promotion of regulatory T-cell differentiation, which may limit the tissue-destructive edge of postoperative inflammation (29, 36). In parallel, SCFAs strengthen barrier competence by increasing mucus production and tight-junction integrity, linking microbial metabolism directly to preventing luminal microbial translocation (37, 38).

#### 2.4.2 Key symbiotic defenders

Several commensals have been proposed as “protective” species not simply by presence, but through defined effector functions relevant to healing. *Faecalibacterium prausnitzii*, a prominent butyrate producer, also secretes a Microbial Anti-inflammatory Molecule (MAM) reported to block epithelial nuclear factor kappa B (NF-κB) signaling and reduce downstream chemokine production (e.g., IL-8) (29). Simultaneously, its metabolites may upregulate tight-junction components such as ZO-1 (25, 35). *Akkermansia muciniphila* may preserve barrier architecture by maintaining mucus-layer turnover and has been linked to MyD88-dependent pathways that enhance IL-22 signaling, a repair-associated axis in epithelial regeneration (23). Emerging candidates such as *Parabacteroides goldsteinii* further suggest that targeted immune regulation—potentially focusing on NF-κB-related circuitry—could represent an additional microbial route to anastomotic protection (27, 39, 40).

Collectively, these observations argue that “beneficial microbiota” should be viewed as a functional state—metabolite production, immunologic regulation, and barrier reinforcement—rather than a simple list of specific species. This distinction becomes central when considering how perioperative dysbiosis may invert these same pathways toward leakage.

## 3 Dysbiosis and the pathological mechanisms of anastomotic leakage

**FIGURE 1:**
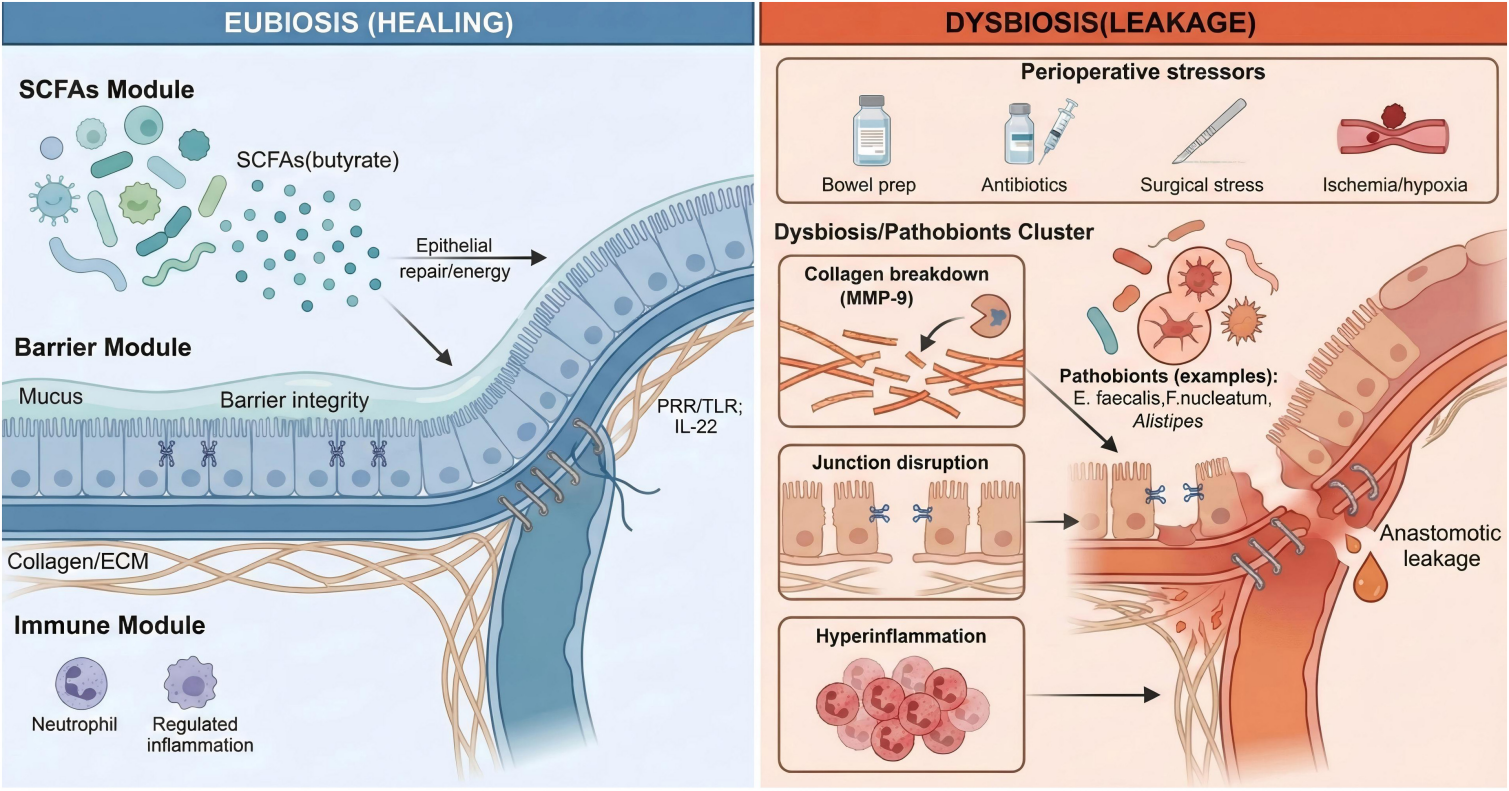
Gut microbiome–driven mechanisms of colorectal anastomotic healing versus leakage. Conceptual schematic comparing eubiosis-associated pro-healing functions with dysbiosis-associated pro-leak mechanisms at the anastomotic interface after colorectal surgery. Under eubiosis, microbial metabolites such as short-chain fatty acids (SCFAs; notably butyrate) support epithelial repair, reinforce barrier integrity (mucus and tight junctions), and maintain a regulated inflammatory milieu. Perioperative stressors can precipitate dysbiosis and pathobiont expansion (examples include *Enterococcus faecalis*, Fusobacterium nucleatum, and *Alistipes*), which converge on collagen/extracellular-matrix breakdown (including host MMP-9 activity), epithelial junction disruption, and hyperinflammation, ultimately increasing the risk of anastomotic leakage (AL).

In physiological eubiosis, the gut microbiome can sustain an anastomosis by regulating inflammation, supporting epithelial repair, and preserving barrier function (16, 17). However, perioperatively, that same ecosystem is readily destabilized into a pro-leak state. Bowel preparation, antimicrobial exposure, operative trauma, and neurohumoral stress can collectively reduce microbial diversity and break down colonization resistance, permitting the expansion of selected taxa and the induction of virulence programs (18, 33, 41). Once enriched, these opportunistic organisms may compromise healing through convergent mechanisms—amplifying local inflammation, disrupting epithelial integrity, and accelerating extracellular matrix breakdown (14, 21, 42)—thereby weakening anastomotic tensile strength and causing leakage (13, 43). The shift from a physiological, pro-healing microbiome to a dysbiotic, tissue-destructive state is illustrated in Figure 1.

### 3.1 The perioperative micro-ecosystem collapse: synergistic disruption by bowel preparation, antibiotics, and surgical stress

Measures intended to reduce infectious risk and optimize operative conditions can unexpectedly destabilize the intestinal ecosystem at the moment an anastomosis requires maximal biological support. Preoperative bowel preparation—particularly mechanical preparation combined with oral antibiotics (surgical bowel preparation, SBP)—has been associated with a deeper loss of microbial diversity and slower microbial recovery than mechanical preparation alone (18, 44, 45). By unevenly depleting obligate anaerobes (including key SCFA-producing taxa), SBP can create an ecological gap that favors the expansion of antibiotic-tolerant facultative organisms such as *Enterococcus* and *Streptococcus* (14, 19, 46). Consistent with a long-lasting disturbance rather than a transient fluctuation, one prospective cohort reported that microbial diversity after bowel resection had not returned to baseline even at 180 days (47).

The operation itself then worsens this vulnerability by reshaping the local biochemical niche. Ischemia–reperfusion injury and hypoxia can shift redox balance and pH, likely selecting for organisms adapted to these stress conditions (20, 31). Furthermore, host stress mediators (e.g., catecholamines) may be sensed by bacteria, triggering quorum-sensing–linked virulence programs that convert otherwise non-invasive commensals into invasive phenotypes (33, 41). Finally, the anatomic disruption of bowel resection appears to induce a more significant and persistent community restructuring than nonresection operations, providing a permissive environment for durable pathogen colonization and activity (48).

### 3.2 Molecular disruption mechanisms of key pathogens

Within a dysbiotic perioperative niche, multiple bacterial taxa can likely target the anastomosis—acting alone or in concert—through distinct but functionally similar virulence programs (49). Despite variability in their “molecular toolkits,” these pathways repeatedly lead to a single biomechanical outcome: destabilization of the extracellular matrix (ECM), with collagen degradation emerging as the dominant lesion that weakens early anastomotic strength (50).

#### 3.2.1 Enterococcus faecalis and collagenolysis

*Enterococcus faecalis* has emerged as a key candidate pathogen associated with AL, with mechanistic insights largely derived from the “dual-hit” model described in animal studies (14). In rat models of anastomosis, virulent strains secrete gelatinase (GelE) and serine protease (SprE), which can directly degrade type I collagen—the principal substrate conferring early anastomotic tensile strength (14, 51). Furthermore, experimental data suggest a second hit: the GelE/SprE system can activate host matrix metalloproteinase-9 (MMP-9), thereby amplifying collagen breakdown through endogenous enzymatic machinery rather than relying solely on bacterial proteolysis (14, 15). These preclinical findings are clinically relevant, as standard intravenous prophylaxis (e.g., cefoxitin) may insufficiently suppress *E. faecalis* at the anastomotic site, offering a plausible explanation for persistent AL rates despite adherence to conventional preventive protocols (14, 52). Observational data further align with this mechanism, reporting higher detection of *E. faecalis* at infectious foci in patients who develop leakage (43, 53).

#### 3.2.2 Fusobacterium nucleatum and immune trafficking

*Fusobacterium nucleatum*—an oral commensal linked to colorectal carcinogenesis—has also been associated with AL. Mechanistic studies in mice and cell lines propose a structured “dual-hit” program that targets epithelial cohesion and immune trafficking (42, 54). The proposed adhesion–invasion axis centers on FadA binding to epithelial E-cadherin, destabilizing cell–cell junctions and promoting E-cadherin internalization with downstream release and nuclear translocation of β-catenin in experimental models (54, 55). In this preclinical model, β-catenin then functions as a transcriptional regulator that induces MMP-9 expression, connecting microbial adhesion with collagen-matrix vulnerability (42). A second, more inflammatory axis was described by Wei et al., who reported activation of the epithelial NOD1/RIPK2/ERK pathway in murine models, with consequent upregulation of IL-1β (21). The resulting neutrophil chemotaxis (“swarming”) may become maladaptive, as excessive neutrophil elastase and MMP release can cause collateral injury on the immature anastomotic matrix (21). Taken together, these findings suggest that *F. nucleatum* may compromise healing not only by weakening epithelial architecture but also by potentially driving a self-reinforcing inflammatory microenvironment that accelerates matrix failure (49, 56).

#### 3.2.3 Pro-inflammatory pathogens

Not all leakage-associated microbes compromise the anastomosis through direct collagenolysis; some appear to undermine healing by shifting the local inflammatory set-point (39, 57). Hajjar et al. identified *Alistipes onderdonkii* as a candidate pathobiont associated with AL. Crucially, fecal microbiota transplantation experiments demonstrated in murine models that communities enriched in *A. onderdonkii* predisposed the animals to a higher postoperative AL incidence (39). Mechanistically, clinical analysis linked *A. onderdonkii* to a preoperative, subclinical mucosal inflammatory state—characterized by increased pro-inflammatory cytokines and chemokines (e.g., MIP-1, MIP-2, MCP-1, IL-17A/F)—which could likely trigger an exaggerated postoperative response and intensify downstream tissue-destructive effect programs, including MMP activity (9, 39). Reports from other disease contexts also suggest that *A. onderdonkii* can behave as a pro-inflammatory organism in a context-dependent manner, requiring cautious interpretation when extrapolating across cohorts and models (57).

#### 3.2.4 The mycobiome and virome

Despite rapid progress in bacterial profiling, the potential contributions of the gut mycobiome and virome to AL remain incompletely defined (19, 46). Limited evidence suggests that fungal organisms such as *Candida albicans* may impair anastomotic repair, either via collagenolytic activity or by forming mixed-species biofilms with bacteria (notably *E. faecalis*), thereby likely amplifying local virulence (58). Importantly, cross-kingdom biofilms are often more tolerant to antimicrobial therapy than single-species biofilms, complicating eradication once established (14, 15). In *Candida* biofilms, the extracellular matrix can impede drug penetration and promote a drug-protected phenotype, and this matrix-dominant architecture may also shield bacterial partners within mixed communities, likely fostering persistent infection and functional “resistance” at clinically achievable concentrations (14). In parallel, bacteriophages could modulate healing indirectly by reshaping key bacterial populations and their functional capacity, although robust peri-anastomotic data are still sparse (17, 59). The divergent roles of these microbial groups—ranging from metabolic support of healing to collagenolytic impairment of the anastomosis—are summarized in Table 1.

**TABLE 1:** Microbiome-associated mechanisms contributing to colorectal anastomotic healing and leakage.

Microbial factor /taxa	Human evidence (clinical association)	Experimental evidence (preclinical/murine models)	Potential effect on anastomosis	Translational implication	Key refs
SCFA-producing commensals (e.g., *Butyrate producers*)	Reduced abundance often observed in feces of AL patients	Butyrate supplies energy to colonocytes and inhibits histone deacetylase (HDAC) to reduce inflammation	Supports epithelial repair and barrier integrity	Rationale for fiber/prebiotic prehabilitation	(25, 48)
*Faecalibacterium prausnitzii*	Lower relative abundance associated with AL and postoperative ileus	Secretes MAM (Microbial anti-inflammatory molecule) which inhibits NF-κB pathway in epithelial cell lines	Anti-inflammatory; reinforces tight junctions	‘	(29, 39)
*Akkermansia muciniphila*	Generally associated with healthy mucosal status	Promotes mucus turnover and epithelial restitution via MyD88/IL-22 signaling in mice	Barrier preservation	Potential probiotic for mucosal healing	(23)
*Enterococcus faecalis*	High relative abundance detected at leak sites and in drain fluid of AL patients	Virulent strains secrete GelE/SprE to degrade collagen; activate host MMP-9 in rat anastomotic models	Collagenolysis (loss of tensile strength)	Supports targeting specific virulence traits	(14)
*Fusobacterium nucleatum*	Enriched in tissues of AL patients; linked to chemo-resistance	Adhesin FadA binds E-cadherin and activates β-catenin/MMP-9; triggers NOD1-dependent inflammation in mice	Junction disruption and hyperinflammation	Highlights need for mucosal (vs. fecal) sampling	(42)
*Alistipes onderdonkii*	Identified in preoperative stool of patients who subsequently developed AL	Transplantation of A. onderdonkii-enriched stool causes AL in mice; induces pro-inflammatory cytokines	Pro-inflammatory “priming” of the anastomosis	Suggests combining microbial and immune biomarkers	(39)
Fungal pathogens (e.g., *Candida albicans*)	Detection in peritoneal fluid associated with severe leakage	Forms mixed-species biofilms with E. faecalis *in vitro*; enhances bacterial virulence and antibiotic tolerance	Persistence of infection; delayed healing	Consideration for antifungal prophylaxis in high-risk groups	(58)

### 3.3 The causation–correlation dilemma: critical appraisal and methodological limitations

Interpreting the microbiome–AL literature demands explicit separation of association from causality, as this distinction continues to define the field’s evidentiary limit. Under modern adaptations of Koch’s postulates, the most compelling causal support derives from interventional animal work—most notably strain-specific inoculation models that reproduce leakage phenotypes, and fecal microbiota transplantation paradigms in which AL-associated communities transmit risk to recipient hosts (14, 39, 60). By contrast, the majority of human datasets remain observational; enrichment or depletion of particular taxa in AL cohorts is informative, yet intrinsically insufficient to determine directionality (61). A persistent “chicken-and-egg” problem therefore remains: dysbiosis may cause impaired healing, but an evolving leak-prone microenvironment (e.g., micro-ischemia and localized inflammation) could also select for dysbiosis, necessitating longitudinal, multi-time-point sampling to resolve temporality.

Several methodological constraints further limit applicability across studies. Sampling strategy is a dominant source of uncertainty: Fecal profiles largely represent luminal communities and may not reflect the mucosa-associated microbiota that directly interfaces with the anastomotic wound (62). Furthermore, margin biopsies have been argued to offer higher mechanistic relevance for AL research (63). Technical variability—spanning 16S rRNA versus metagenomic approaches and variable bioinformatic pipelines—can yield conflicting taxonomic signals even when cohorts are clinically similar (64, 65). Clinical confounding is equally difficult to control, given inter-patient differences in diet, comorbidities, and medication exposure that can independently alter microbial ecology and host inflammatory tone (18). Many reports are also underpowered, particularly for subgroup analyses, and the lack of large prospective multicenter cohorts continues to prevent robust validation.

These limitations argue for a more structured next generation of study designs rather than gradual expansion of cross-sectional profiling. Priority should be given to paired mucosal and luminal sampling across multiple perioperative time points, coupled with integrated multi-omics and analytical frameworks capable of causal inference rather than descriptive clustering alone. Such rigor would sharpen biological understanding, clarify the boundaries of current claims, and better position microbiome-targeted strategies for reliable clinical application.

## 4 Predictive biomarkers for anastomotic leakage risk stratification

Given the high morbidity associated with AL—and the narrow window in which early intervention may still be effective—robust biomarkers for preoperative or very early postoperative risk assessment have become a central clinical priority. Conventional risk scores remain useful for baseline triage, yet their predictive ability is often insufficient when applied to individual decision-making in diverse surgical populations. Against this backdrop, the gut microbiome and its two-way interaction with the host offer a mechanistically sound basis from which a more informative biomarker class may be obtained. Current candidates broadly fall into three domains: Microbe-derived signatures, metabolite-level readouts, and host-response markers, with multi-omic integration—potentially augmented by artificial intelligence—representing the most plausible route toward clinically useful prediction.

### 4.1 DNA-based biomarkers: from diversity to microbial fingerprints

High-throughput sequencing of gut microbial composition remains the dominant strategy for microbiome-informed AL risk prediction. At the community level, α-diversity has yielded inconsistent results—some cohorts link AL with reduced diversity, whereas others report higher α-diversity, likely reflecting pathogen growth and community disruption rather than ecological stability (45, 46, 56). In contrast, β-diversity more consistently distinguishes AL from non-AL patients, supporting the idea that pattern-based community changes—not a single numerical measure—better capture risk-related dysbiosis (56, 66).

Efforts have therefore shifted toward taxa-based signatures, with multiple studies agreeing on a profile marked by higher levels of genera such as *Fusobacterium, Enterococcus*, and *Alistipes* (among others) and lower levels of taxa such as *Parabacteroides, Faecalibacterium* (notably *F. prausnitzii*) and *Lactobacillus* (27, 39, 43, 67). Sampling location significantly affects how results are interpreted: mucosal biopsies or margin swabs are often said to better reflect the microenvironment that directly interacts with the anastomosis than fecal samples (33, 62). Technology choice further affects predictive accuracy, as metagenomic shotgun sequencing can provide species or strain-level information and functional gene content (e.g., virulence and resistance genes) that 16S rRNA sequencing usually cannot (15, 65). In one report, a species-level classifier from metagenomic data achieved an area under the curve (AUC) of 0.92 for AL prediction, outperforming a 16S-based model (AUC 0.76), showing the added value of more detailed taxonomic and functional information (68).

Beyond compositional profiling, metagenomic sequencing also enables direct detection of functionally relevant DNA sequences—most notably virulence genes and antimicrobial resistance determinants—that may more specifically reflect anastomotic risk than taxon abundance alone. Virulence gene targets identified in prior mechanistic work, such as gelE and sprE (encoding the *Enterococcus faecalis* collagenolytic machinery) and fadA (encoding the Fusobacterium nucleatum adhesin), represent logical candidates for preoperative risk stratification, as their presence in mucosal or luminal samples could indicate enrichment of strains with direct tissue-destructive capacity (14, 15, 54). Metagenomic detection of antimicrobial resistance genes may also complement prophylaxis planning, given that conventional regimens do not reliably cover all high-risk lumenal pathogens at the anastomotic site (14, 52).

The predictive value of DNA-based biomarkers is also substantially affected by sampling timing and specimen type. Preoperative sampling captures the baseline community before dysbiosis is induced by surgical preparation, potentially identifying patients who enter the perioperative period with an already vulnerable microbial ecology; by contrast, early postoperative sampling may detect pathobiont expansion triggered by operative trauma and antibiotic exposure, offering a complementary and dynamically updated risk signal (66, 68). Integrating preoperative and early postoperative DNA profiles—rather than relying on a single time point—may therefore yield a more complete picture of the biological trajectory toward anastomotic failure or successful healing.

### 4.2 Metabolomics and multi-omics signatures: SCFAs, bile acids, and integrated profiles

Because microbial effects are ultimately exerted through biochemical outputs, metabolite readouts may track pathophysiology more directly than composition alone (65, 69). Among candidate metabolites, reduced short-chain fatty acids—particularly butyrate—have been identified as a likely risk signal when detected in feces or blood before or early after surgery, consistent with the proposed role of SCFAs in supporting mucosal repair (2, 37). Supporting this concept, Kohn et al. reported that SCFA levels dropped significantly in the intestinal resection subgroup, aligning microbial disruption with functional metabolic loss (48). Dysbiosis-related shifts in bile acid profiles also deserve attention, as certain secondary bile acids such as deoxycholic acid are described as pro-inflammatory and cytotoxic, features that likely compromise healing (70, 71). Beyond these established classes, untargeted metabolomics has begun to identify additional candidates (e.g., selected amino acids, lipids, and polyamines), although their specificity and causal connection remain to be defined (72).

Single-layer biomarkers are unlikely to fully capture a process as complex as AL, which emerges from host–microbiome interaction rather than microbial abundance alone (41, 73). The field is therefore moving toward integrated multi-omics models that link microbial structure to functional metabolites and then to host transcriptional, proteomic, or immune phenotypes, with the goal of building risk-relevant networks rather than reporting disconnected associations (74, 75). If validated across cohorts, this systems-level approach could support more generalizable prediction tools than any single taxon or metabolite measured alone (76).

### 4.3 Host-derived biomarkers: mucosal cytokines and circulating immune cells

Because AL emerges from host–microbiome interaction, host-response readouts can complement microbial or metabolite signatures and may better reflect the preoperative “immune set-point” of the anastomotic field (9, 41). In the work by Hajjar et al., patients who later developed AL exhibited an already activated, subclinical inflammatory state in colonic mucosa before surgery, marked by higher levels of cytokines/chemokines such as MIP-1, MIP-2, MCP-1, and IL-17A/F (39). This observation likely shows that risk is not solely determined by intra- or postoperative events; instead, baseline mucosal immune tone may prime an exaggerated response to surgical injury, with a secondary increase in tissue-degrading programs (including MMP activity) that can compromise repair.

A clinically useful application is that this mucosal inflammatory pattern may be partially reflected in peripheral blood. Higher preoperative circulating neutrophil and monocyte counts have been reported among AL patients, raising the possibility of a simple, non-invasive screening tool for risk identification (39, 77, 78). In contrast, postoperative C-reactive protein (CRP)—although widely used and helpful for ruling out AL when low—often has low specificity because it rises across diverse inflammatory complications, limiting its value as a stand-alone “rule-in” marker (6, 79).

### 4.4 Integrating multi-omics with AI: toward actionable prediction

No single biomarker class (microbial, metabolic, or host-derived) is likely to capture the full biology of AL, given that each readout samples only one layer of a multi-factorial process (73). A more reasonable approach is to integrate clinical variables with multi-omics data—metagenomic features (species/strain calls, virulence or resistance genes), metabolomic profiles, and immune mediators—using machine learning approaches designed to model non-linear interactions (74, 80). Conceptually, an “ideal” model would include standard clinical risk factors alongside preoperative mucosal swab/biopsy metagenomics and blood-based metabolite/cytokine signals, then output a patient-specific risk score that can be updated as new perioperative data become available (13, 81).

In practice, such models should function as decision support rather than a replacement for surgical judgment. A high-risk output could justify intensified surveillance, earlier imaging triggers, or selective diversion in uncertain cases, aligning biological risk with operative planning and postoperative monitoring. Notably, a recent multicenter effort cited in this manuscript used an ensemble “meta-model” (including CatBoost and LightGBM) that reportedly achieved an internal-validation F1 score of approximately 87% for AL prediction, substantially outperforming traditional logistic regression and single-modality classifiers (82). This performance gap supports the idea that AL risk is better approximated by modeling host–microbiome interactions than by relying on linear clinical predictors alone. A summary of candidate biomarkers across these domains, including their potential utility and current limitations, is provided in Table 2.

**TABLE 2:** Candidate biomarkers for microbiome-informed risk stratification of colorectal anastomotic leakage.

Domain	Sample type	Candidate features (examples)	Timing	Potential utility	Key limitations	Key refs
Microbe-derived signatures (16S/amplicon)	Stool	Community pattern shifts; taxa-based signatures (e.g., enrichment of leak-associated genera)	Preop/early postop	Noninvasive screening; hypothesis generation	Stool may poorly reflect mucosa/anastomotic niche	(56)
Mucosa-associated microbiome	Mucosal biopsy/swab	Margin-/mucosa-proximal taxa and functions	Preop/periop	Higher mechanistic proximity to wound interface	Invasive; sampling standardization needed	(66)
Shotgun metagenomics (functional)	Stool or mucosa	Species/strain resolution; virulence/resistance genes; improved classifier performance reported in some studies	Preop	Better prediction and interpretability (function-level)	Cost, turnaround time; pipeline heterogeneity	(68)
Metabolite readouts	Stool/serum	SCFAs (notably butyrate depletion); bile acid profile shifts; untargeted metabolite panels	Preop/early postop	Functional readout closer to pathophysiology	Diet/medication confounding; assay harmonization	(71)
Host-response markers	Mucosa + blood	Preop mucosal cytokine/chemokine activation; circulating neutrophils/monocytes	Preop	Captures immune “set-point” complementing microbiome	Low specificity alone; best used in integration	(77)
Integrated multi-omics + ML	Combined	Clinical + metagenomics + metabolomics + immune mediators → risk score	Preop ± dynamic update	Individualized stratification; tiered management support	External validation and interpretability required	(82)

## 5 Perioperative intervention strategies to modulate the gut microbiome

Recognizing the gut microbiome as a mechanistic contributor to AL has sharpened interest in perioperative strategies that adjust the intestinal ecosystem—supporting SCFA-producing commensals while controlling opportunistic pathogens at the anastomotic site. This transition shifts prevention from a purely technical or antimicrobial problem toward a biologically informed modulation of host–microbe dynamics. Accordingly, a diverse but rapidly expanding intervention set is being evaluated, spanning dietary prehabilitation, microbial supplementation (probiotics/prebiotics/synbiotics), targeted antimicrobial approaches, and full community recovery strategies such as fecal microbiota transplantation. Key perioperative strategies, along with their proposed mechanisms and implementation considerations, are detailed in Table 3.

**TABLE 3:** Perioperative microbiome-directed strategies for prevention of colorectal anastomotic leakage: proposed mechanisms, evidence, and implementation considerations.

Strategy	Intended microbiome effect	Timing	Evidence summary (qualitative)	Safety/practical notes	Outcome signal to report	Key refs
High-fiber dietary prehabilitation	Restore diversity and SCFA production; strengthen barrier resilience	Preop (short course)	Strong preclinical signal; human evidence heterogeneous	Must define dose/duration/adherence	AL, SSI, ileus, Length of Stay (LOS)	(34)
Probiotics (single strain)	Add beneficial functions (SCFA/immune modulation)	Periop	Some RCT signals for recovery/ infections	Caution in immunocompromised; product QA	Overall infections; AL often inconsistent	(92)
Synbiotics (probiotic + prebiotic)	Promote engraftment and SCFA output	Periop	Meta-analyses suggest lower overall infections	Formulation heterogeneity; dosing varies	SSI and other infections; AL as prespecified endpoint	(92)
Oral antibiotics + bowel prep (selected regimens)	Reduce luminal pathogen burden	Preop	Reported SSI reduction; possible AL benefit in some analyses	Stewardship; regional variability	SSI, AL, C. difficile	(96)
Selective digestive decontamination (SDD)	Suppress aerobes/yeasts while sparing anaerobes	Periop	Cohort/matched analyses suggest lower AL/SSI in some protocols	Resistance ecology; protocol standardization	AL, SSI	(94)
Fecal microbiota transplantation (FMT)	Full community restoration toward “pro-heal” state	Investigational (preop)	Strong mechanistic plausibility; limited AL-specific human trials	Donor screening/regulatory constraints	Feasibility + safety; AL exploratory	(93)

### 5.1 Nutritional strategies: high-fiber prehabilitation

Diet represents one of the most powerful external factors of microbiome structure and function, making it an intuitively feasible strategy for perioperative risk modification (83, 84). Preclinical work consistently indicates that a high-fat, low-fiber “Western” dietary pattern can cause dysbiosis, suppress SCFA production, and compromise barrier integrity—changes that align with higher AL rates and mortality in murine models (34, 50, 85). Notably, the microbiome appears highly adaptable: Even after prolonged Western-diet exposure, a brief preoperative switch (approximately 2–7 days) to a high-fiber, low-fat regimen can restore microbial diversity, augment butyrate and other SCFAs, and significantly improve anastomotic healing and postoperative survival (34).

Translational evidence in humans, however, remains less definitive. Cohort studies evaluating habitual preoperative fiber intake have not uniformly demonstrated a protective association with AL, a gap that likely reflects dietary complexity, inter-individual variability in fiber responsiveness, and the limitations of retrospective dietary assessment (86). Consequently, the field needs prospective trials that define “high-fiber prehabilitation” as a standardized, quantifiable intervention—defined by dose, duration, and adherence metrics—so efficacy can be evaluated against clinically meaningful endpoints.

### 5.2 Microbial supplementation: probiotics, prebiotics, and synbiotics

Microbial supplementation—either by introducing beneficial organisms or by supplying substrates that specifically support them—offers a straightforward approach to adjusting the microbiome during the perioperative period. In a large randomized controlled trial (RCT) of patients having curative colorectal cancer surgery, the administration of *Clostridium butyricum* (CBM588) during this time was associated with accelerated bowel recovery after surgery (e.g., earlier gas passage), fewer infections (including intra-abdominal infections), and an improved immune profile, evidenced by increased T-cell counts in peripheral blood (87). Other clinical studies also suggest that mixtures containing *Lactobacillus* and *Bifidobacterium* can change the community structure after surgery, reduce potentially harmful taxa like *Enterobacteriaceae* and *Pseudomonas* and strengthen the mucosal barrier function (67, 88). Prebiotics (e.g., fructo-oligosaccharides and inulin) work mainly by feeding SCFA-producing commensals, thus increasing butyrate production and the later pro-repair effects described earlier (89). Synbiotics combine both methods and, at least in theory, aim to improve growth and metabolic activity through paired strain-substrate design (90).

Across meta-analyses, probiotic or synbiotic treatments seem to reduce overall infections after colorectal surgery—especially lung and urinary tract infections (91, 92). However, when AL is evaluated as a specific outcome, the preventive effect has not been statistically significant (reported RR 0.83, 95% CI 0.47–1.48), meaning that “general” microbiome improvement may not be enough to counteract the highly localized, collagenase-driven process of anastomotic failure (92). Regarding safety profiles, live beneficial microbes are well accepted in most patients, but potential risks of bacteremia remain a clinical concern for patients with compromised immune systems or who are critically ill, so careful patient selection and monitoring are required for clinical implementation (59, 92).

### 5.3 Antimicrobial strategies: decontamination and antibiotic choice

The guiding concept of antimicrobial modulation is to target and reduce high-risk pathogens in the lumen while preserving anaerobic communities that support SCFA production and barrier function (46, 93). Conventional single-dose intravenous prophylaxis, although essential, has limited influence on lumenal pathogens such as *Enterococcus faecalis* and does not reliably prevent their attachment to the anastomosis (14, 52). Selective digestive decontamination (SDD) enhances this approach by administering non-absorbable, broad-spectrum agents orally or via enemas to clear aerobic Gram-negative bacteria and yeasts while largely sparing strict anaerobes; several cohort studies and matched analyses report lower rates of AL and surgical-site infection (SSI), with acceptable cost profiles under tightly controlled protocols (94, 95). Even so, the optimal SDD cocktail, the subset of patients who truly benefit, and the long-term impact on microbial balance in terms of antibiotic resistance remain unresolved questions that require large randomized trials (59).

Although further RCTs are needed, current data allow some tentative practice guidelines and highlight areas of controversy. First, guideline-compliant intravenous prophylaxis that covers *Enterobacterales* and anaerobes—such as cefazolin plus metronidazole or cefoxitin—remains a valid starting point, and recent multicenter microbiologic surveillance does not support regular use of stronger agents in unselected patients (96). Second, multiple network meta-analyses suggest that combining intravenous and short-course oral antibiotics (for example, neomycin plus metronidazole given with mechanical bowel preparation) reduces SSI and probably AL compared with intravenous prophylaxis alone, although effect sizes vary and practice is variable across regions (44, 96). Third, simply choosing agents with broader *Enterococcus* coverage has not consistently improved outcomes: in a large Swiss cohort, amoxicillin–clavulanate (with *E. faecalis* activity) was associated with higher SSI risk than cefuroxime plus metronidazole, and other observational work links cefoxitin prophylaxis to more cephalosporin-resistant flora in subsequent infections (97). Taken together, these findings argue for a microbiologically rational but conservative strategy—robust Gram-negative and anaerobic coverage for all, cautious use of SDD or strong oral treatments in clearly defined high-risk groups, and ongoing local surveillance to adjust specific drug choices rather than a complete change to ever broader perioperative antibiotics.

### 5.4 Microbiome reconstruction: fecal microbiota transplantation

Fecal microbiota transplantation (FMT) currently represents the most comprehensive approach to microbiome restoration, aiming to re-establish a diverse, functionally healthy ecosystem by transferring stool-derived communities from a screened healthy donor to the recipient (98). Its clinical effectiveness in recurrent *Clostridioides difficile* infection provides proof-of-concept that significant ecological repair is achievable in humans, supporting the biological possibility of perioperative applications (37, 98). In the context of AL, preclinical work by Hajjar et al. used FMT in mouse models to show that microbiota from patients who developed AL can pass on a “pro-leak” trait, while microbiota from patients with uncomplicated healing appears more protective—an observation that strengthens the case for a direct microbiome role in anastomotic integrity (39).

From a practical research view, a likely short-term model would reserve FMT for carefully chosen, high-risk patients with clear preoperative dysbiosis, with the goal of replacing a permissive “pro-leak” community with a “pro-heal” structure before anastomotic construction (13, 59). That said, implementation remains limited by major unresolved issues, including: Donor screening and standardization, optimal timing and delivery method, safety in patients with weakened immune systems, ethical concerns, and evolving regulatory rules (98, 99). At present, large-scale human trials evaluating FMT specifically for AL prevention have not been published, and the strategy should be framed as experimental rather than practice-ready (99).

## 6 Critical assessment: translational barriers and heterogeneity of perioperative microbiome strategies

Overall, perioperative microbiome-focused strategies are biologically compelling, yet they have not matured into a reliable, standard set of methods suitable for routine colorectal practice (61, 99). A common issue is the weakening—or complete loss—of effect when interventions that work well in controlled laboratory settings are applied to diverse human groups (100, 101). Even where clinical benefits are observable (e.g., reduced overall infectious morbidity), the evidence for AL prevention often remains inconsistent, underscoring that anastomotic failure may require more specific spatial and functional changes than general microbiome improvement can provide (91, 92).

A second limit is patient-to-patient differences. Baseline community structure, host genetics, dietary factors, and immune state vary markedly between individuals, likely explaining why identical interventions (including specific probiotic mixes) lead to different biological and clinical results (18, 65). This finding challenges the common one-size-fits-all approach and supports a shift toward tailored or personalized plans anchored in preoperative host–microbiome patterns (102).

Operational translation introduces additional challenges. Integrating microbiome interventions into surgical workflows requires resolution of practical questions—strain selection, dosing, timing, duration, and endpoints—alongside broader considerations such as regulatory pathways for live biological therapies, cost-effectiveness, and acceptance by surgeons and patients (59, 103). Accordingly, while the current toolkit offers a credible starting point for perioperative microbial modulation, precise and long-lasting AL prevention will likely depend on rigorously designed, adequately powered trials coupled to mechanistic biomarkers that identify who should receive which intervention, and when.

## 7 Future outlook

Over the next 5–10 years, progress will likely depend on moving from broad, observational “microbiome modulation” to interventions that are based on clear mechanisms, tailored to individual patients, and practical for perioperative care pathways (102). This shift is motivated by two key observations: first, AL appears to reflect highly localized host–microbe interactions at the anastomotic interface; second, differences between individuals in baseline gut ecology and immune state can weaken the impact of uniform protocols when tested in real-world groups. Accordingly, next-generation strategies should prioritize actionable microbial functions (virulence programs, collagen-breaking ability, metabolite loss) rather than just taxonomic names (102).

### 7.1 Engineered live biotherapeutics

Synthetic biology offers a plausible way to create “sense-and-respond” live biotherapeutics that act as on-site effectors rather than passive colonizers (103, 104). In principle, a safe probiotic base (e.g., *E. coli* Nissle 1917 or *Lactococcus lactis*) could be genetically modified to detect early signs of disease in the anastomotic microenvironment—hypoxia-related metabolites or inflammatory mediators—and then release a therapeutic substance only upon specific triggers (103, 105). Candidate substances include locally acting anti-inflammatory mediators (e.g., IL-10), epithelial repair factors (e.g., trefoil factors), inhibitors similar to tissue inhibitors of metalloproteinases (TIMPs) to reduce excess MMP activity, and narrowly targeted bacteriocins aimed at key collagen-breaking or pro-inflammatory taxa, thereby preserving beneficial anaerobes while suppressing specific threats (103, 106). The main challenge for translation remains biosafety (horizontal gene transfer, uncontrolled persistence) and reliable delivery/establishment at the anastomotic site—limits that will determine whether this approach remains experimental or achieves clinical utility (101).

**FIGURE 2:**
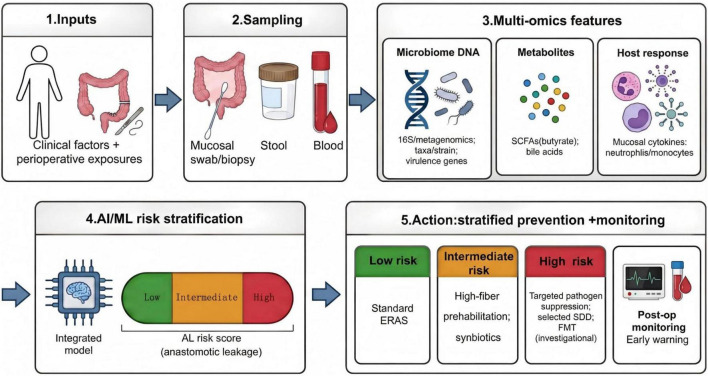
Conceptual workflow for microbe-informed risk stratification and prevention of anastomotic leakage. Proposed perioperative framework integrating clinical variables with multi-omics data to estimate individualized AL risk and guide tiered management. Sampling may include mucosal swabs/biopsies and stool for microbiome profiling (16S rRNA sequencing or metagenomics) alongside metabolite readouts (e.g., SCFAs and bile acids) and host-response markers (e.g., mucosal cytokines and circulating neutrophils/monocytes). Features are integrated using AI/machine-learning models to generate a low/intermediate/high risk score, which can inform stratified preventive strategies (e.g., standard ERAS, dietary fiber prehabilitation and synbiotics, selected pathogen-suppressive approaches, and investigational microbiome reconstruction) with enhanced postoperative monitoring for early warning.

Personalized perioperative microbiome management. A one-size-fits-all perioperative regimen (uniform bowel preparation and antibiotics for all patients) is increasingly difficult to justify given variability in baseline dysbiosis, pathogen enrichment, and host immune states (61). A more coherent model would pair rapid preoperative multi-omics sampling (mucosal swab metagenomics plus blood metabolite/cytokine profiling) with AI-driven risk stratification, then match interventions to the dominant biological issue (10, 65). A proposed conceptual framework integrating these multi-omics inputs into a tiered clinical decision pathway is presented in Figure 2. Low-risk patients could remain on standard ERAS pathways, intermediate-risk patients could receive targeted synbiotics or short-course dietary prehabilitation to restore SCFA-producing capacity (34, 89), high-risk patients might need more intensive pathogen-suppressive strategies (e.g., SDD for virulent *E. faecalis* enrichment) or, in extreme microbial imbalance, consideration of donor-derived community restoration such as FMT as an investigational option (37, 98).

### 7.2 AI-enabled postoperative monitoring and early warning

Anastomotic healing is dynamic, and the perioperative microbiome evolves in parallel; static preoperative prediction therefore risks missing late shifts toward a “pro-leak” path (66). A future monitoring framework could integrate ongoing physiology (vital signs, routine labs), serial microbial and metabolite measurements from stool or stoma output, and—conceptually—local microenvironment signals (pH, oxygen tension, lactate) captured by degradable or implantable sensors to generate continuously updated leak-risk estimates (82, 103). If validated, such systems could identify actionable deviations (e.g., abrupt pathogen expansion or SCFA depletion) before overt clinical deterioration, thereby widening the window for preventive imaging, antibiotic adjustment, or escalation of surveillance (76). Cost and feasibility remain significant—daily metagenomic profiling is not currently scalable—so near-term success will likely depend on cheaper alternative biomarkers or streamlined assays that retain predictive value without imposing excessive resource demands (79, 100).

### 7.3 Key scientific questions for the next decade

Achieving microbiome-informed prevention of anastomotic leakage will require more than small-scale association studies; the field now needs mechanistic precision, standardized intervention logic, and usable clinical infrastructure. The following questions, if answered rigorously, would turn a promising idea into a usable perioperative strategy.

#### 7.3.1 From taxa to function (and beyond bacteria)

Future work should define “pro-heal” versus “pro-leak” states at the level of microbial functions—metabolic pathways, virulence programs, and strain-level attributes—rather than relying on species lists alone. This agenda also requires clear inclusion of the mycobiome, virome, and phage ecology, as multi-kingdom interactions may significantly affect the anastomotic niche yet remain not well understood.

#### 7.3.2 Causality, not correlation

Observational multi-omics signals must be evaluated in models with higher human relevance, including gut organoids and humanized systems that allow controlled testing of candidate microbes, metabolites, and host pathways. Without this causal foundation, biomarker discovery and intervention design will continue to lead to hard-to-repeat or context-specific findings.

#### 7.3.3 Personalized intervention rules

For probiotics/synbiotics and nutritional prehabilitation, the field should agree on practical parameters—strain combinations, dosing, timing, and duration—then test these in sufficiently large RCTs with clearly defined endpoints, including AL as a prespecified outcome. A parallel priority is to define clearly how baseline microbial signatures determine assignment to each intervention tier, so “personalization” becomes a reliable system rather than a general description.

#### 7.3.4 Biosafety and delivery of engineered live biotherapeutics

Engineered probiotics will only be credible if biosafety concerns (horizontal gene transfer, unintended persistence, uncontrolled expansion) are addressed with strong safety measures and predictable pharmacokinetics. Delivery technologies must also ensure viability and site-specific activity at or near the anastomosis, where the relevant host–microbe interactions occur.

#### 7.3.5 Robust and interpretable AI

Predictive models must work well for different institutions, regions, and populations, avoiding loss of accuracy when used outside the training group. Interpretability also matters: clinicians will not use risk scores in practice unless the model’s key factors are clear enough to support credible, protocol-based actions.

If these scientific and practical gaps are closed, microbiome-based perioperative care could likely evolve from experimental additions into a structured, targeted system that reduces AL while considering feasibility and safety limits.

## 8 Conclusion

The integration of the gut microbiome into the pathophysiology of colorectal anastomotic leakage represents a fundamental paradigm shift, moving the field beyond a purely mechanical view of surgical failure. As synthesized in this review, cumulative evidence now defines the perioperative period as a critical ecological window where the loss of colonization resistance and the expansion of collagenolytic pathobionts—such as Enterococcus faecalis, Fusobacterium nucleatum, and Alistipes onderdonkii—can compromise an otherwise technically sound anastomosis. Conversely, the preservation of a metabolically active, SCFA-producing commensal community appears essential for regulating local inflammation, maintaining barrier integrity, and fueling epithelial repair.

While the biological plausibility of microbiome-informed care is robust, clinical translation has reached a pivotal juncture. Current intervention strategies, ranging from dietary prehabilitation and synbiotics to selective decontamination and fecal microbiota transplantation, offer promising therapeutic avenues but are currently limited by effect heterogeneity and a lack of standardization. This variability underscores that a “one-size-fits-all” approach is insufficient; individual differences in baseline microbial ecology and host immune tone necessitate a transition toward precision surgery.

Ultimately, the future of AL prevention lies in bridging the gap between molecular discovery and operative decision-making. By leveraging integrated multi-omics and artificial intelligence to stratify biological risk, the field can move toward personalized perioperative modulation—treating the microbiome not merely as a bystander, but as a modifiable partner in healing. Realizing this potential will require rigorously designed clinical trials that validate causal biomarkers and targeted therapies, thereby transforming the gut microbiome from an invisible threat into a powerful tool for improving oncologic and surgical outcomes.

## Data Availability

The original contributions presented in the study are included in the article/supplementary material. Further inquiries can be directed to the corresponding author.
